# DEPTOR regulates nucleus pulposus cell senescence through the mTORC1/S6K1/ATG1 pathway to alleviate intervertebral disk degeneration

**DOI:** 10.1038/s41420-025-02819-9

**Published:** 2025-11-17

**Authors:** Hui Lu, Zhiming Liu, Yan Wang, Shuo Han, Xianjuan Zhang, Rong Liu, Yusi Gao, Hualei Liu, Hao Tao, Xuexiao Ma, Zhu Guo

**Affiliations:** 1 Department of Spinal Surgery, The Affiliated Hospital of Qingdao University, Qingdao, China; 2 Qingdao Medical College, Qingdao University, Qingdao, China; 3 Wuhan Liu Sanwu Bone Injury Hospital of Traditional Chinese Medicine, Wuhan, China; 4 Department of Clinical Laboratory, The Affiliated Hospital of Qingdao University, Qingdao, China; 5 Department of Orthopedics, Institute of Medical Innovation and Transformation, Puren Hospital affiliated to Wuhan University of Science and Technology, Wuhan, China; 6 Department of Operating room, The Affiliated Hospital of Qingdao University, Qingdao, China

**Keywords:** Cell biology, Autophagy

## Abstract

This study aimed to determine the molecular mechanisms by which the DEP domain-containing mTOR-interacting protein (DEPTOR) regulates the senescence of nucleus pulposus (NP) cells (NPCs), alleviating intervertebral disk degeneration (IDD). This study investigated how DEPTOR regulates the mechanistic target of rapamycin complex 1 (mTORC1)/S6 kinase beta-1 (S6K1)/autophagy-related gene 1 (ATG1) pathway to regulate senescence-associated secretory phenotype (SASP) and cellular autophagy in NPCs. Isobaric tags for relative and absolute quantitation was used to measure the differences in protein expression between degenerated and normal intervertebral disk tissues. Western blotting and immunofluorescence were used to quantify DEPTOR levels in NP tissues. DEPTOR was overexpressed in vitro, and changes in autophagy and SASP were monitored to determine its effects on NPCs. Moreover, lentiviral overexpression of S6K1 (LV-S6K1) and siRNA-mediated knockdown of ATG1 (ATG1-ShRNA) in both in vitro and in vivo models were used to verify whether DEPTOR stimulates autophagy in NPCs via ATG1 and inhibits SASP through S6K1. The results demonstrated that degenerated intervertebral disks had lower DEPTOR levels. Matrix metalloproteinases, inflammatory cytokines, chemokines, and aging-related proteins were downregulated when DEPTOR was overexpressed in NPCs. Furthermore, autophagic activity was stimulated, SASP secretion was inhibited, and extracellular matrix synthesis was increased. ATG1 knockdown decreased the capacity of DEPTOR to promote cellular autophagy and inhibit SASP, whereas S6K1 overexpression diminished DEPTOR-mediated SASP inhibition. DEPTOR attenuates IDD by inhibiting SASP secretion via the mTORC1/S6K1 pathway and promoting autophagy in NPCs via the mTORC1/ATG1 pathway.

## Introduction

Low back pain (LBP) is a common ailment that has adverse effects on patients and society [1, 2]. In the USA, approximately 25% of adults have reported experiencing LBP lasting for at least 24 h within the past 3 months, and the lifetime prevalence of LBP is estimated to be as high as 80% [3, 4]. Numerous studies have indicated an association between LBP and intervertebral disk degeneration (IDD) [5, 6]. Although it is a major contributor to several degenerative lumbar spine diseases, clinical treatments and effective interventions for IDD are insufficient [7].

The pathophysiology of IDD is reported to be significantly influenced by the senescence of nucleus pulposus (NP) cells (NPCs) [8]. The senescence-associated secretory phenotype (SASP) is activated in degenerated intervertebral disks, indicating an increase in senescent NPCs. The specific manifestations are as follows: the reduction of functional cells within the disk; breakdown of the extracellular matrix (ECM); initiation of inflammation; and aberrant release of metalloproteinases, proinflammatory factors, and chemokines. IDD advances rapidly because of the inflammatory response [9, 10]. SASP inhibition in NPCs is an important strategy for reversing NPC senescence and alleviating IDD progression because NPC senescence is considered a major pathological factor in IDD [11, 12].

The mechanistic target of rapamycin (mTOR), a highly conserved serine/threonine kinase, is present in two different complexes: mechanistic target of rapamycin complexes 1 and 2 (mTORC1 and mTORC2, respectively) [13]. Studies have demonstrated that during cellular senescence, the mTOR signaling pathway regulates several biological processes, such as mRNA transcription, ribosomal biosynthesis, protein synthesis, apoptosis, autophagy, and inflammatory reactions. Cellular senescence is predominantly modulated by mTOR [14]. By phosphorylating mRNA transcription factors and proteins involved in protein synthesis, mTORC1 regulates downstream effector proteins, including ribosomal protein subunits and p70/S6 kinases (S6Ks). Phosphorylation of p70/S6Ks increases ribosomal synthesis, which in turn promotes the release of senescence-associated factors [15]. Furthermore, autophagy-related gene 1 (ATG1) is directly phosphorylated and inhibited by mTORC1, which prevents the formation of a kinase complex comprising ULK1, Atg13, and FIP200. This process prevents autophagosome formation. Autophagic degradation efficiency is reported to decline with age, and an Akt-independent protein called DEP domain-containing mTOR-interacting protein (DEPTOR) stimulates autophagy by directly inhibiting mTORC1 [16]. mTOR, raptor (mTOR-related regulatory protein), and mLST8 are the three primary components of mTORC1. Raptor can be used as a stand-in marker for mTORC1 expression [17]. mTORC1 and mTORC2 share the structural protein DEPTOR, which inhibits them endogenously [13]. This implies that S6 kinase beta-1 (S6K1) and ATG1, which affect cellular autophagy, influence cellular senescence. DEPTOR regulates the mTORC1/S6K1/ATG1 pathway, which in turn regulates cellular senescence. Despite studies on the function of DEPTOR in different tissues and organs, its precise function in intervertebral disks has not been explored [18, 19]. Therefore, this study aimed to determine the molecular mechanisms by which DEPTOR regulates cellular autophagy and SASP in NPCs via the mTORC1/S6K1/ATG1 pathway and its association with IDD.

## Results

### DEPTOR is reduced in degenerated intervertebral disks

**Fig. 1:**
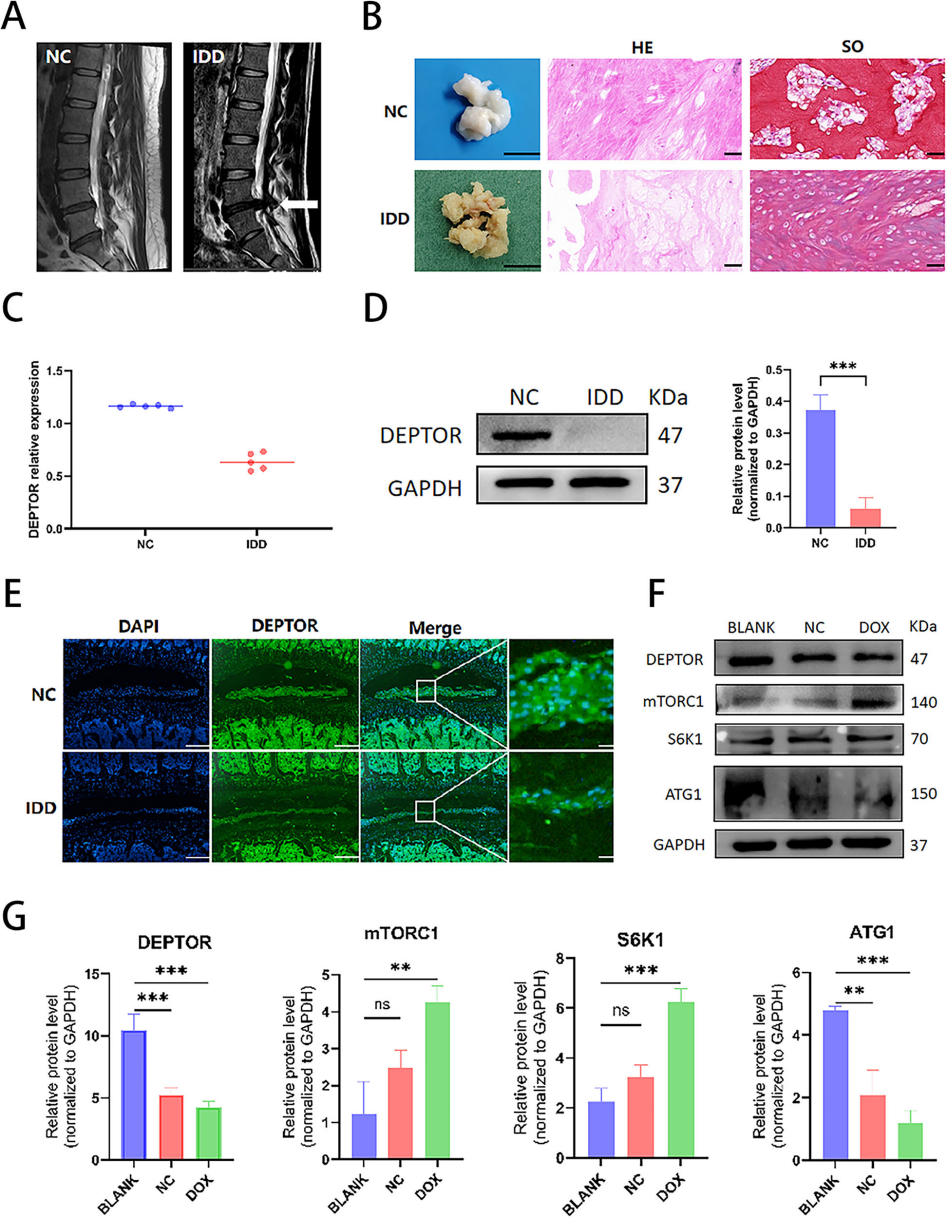
Expression of DEPTOR in normal and degenerated human and rat intervertebral disks and expression of mTOR pathway–related proteins in senescent nucleus pulposus cells (NPCs). **A** T2-weighted magnetic resonance images of normal (left) and degenerated (right) human disks. White arrows indicate degenerated disks. **B** Overview of donor nucleus pulposus (NP) tissue obtained during discectomy; scale bar: 1 cm. Hematoxylin and eosin (HE) and safranin O (SO) staining of degenerated human NP tissue from the intervertebral disk (right); scale bar: 200 µm. **C** Quantitative proteomics analysis via isobaric tags for relative and absolute quantitation (iTRAQ) showing DEPTOR protein levels in human intervertebral disk tissue. **D** Western blot (WB) detection and quantitative analysis of DEPTOR protein in human NP tissue. **E** DEPTOR detection in rat NP tissue; scale bar: 200 µm. **F**, **G** DOX-induced senescence in NPCs with WB detection and quantitative analysis of DEPTOR, mTORC1, S6K1, and ATG1. Data are presented as means ± standard deviations; ns nonsignificant; *p < 0.05; **p < 0.01; ***p < 0.001; ****p < 0.0001.

Magnetic resonance T2-weighted imaging (T2WI) revealed different signal intensities for normal and degenerated intervertebral disks, with the former displaying noticeably lower signal intensities than the latter (Fig. 1A). The NP tissue of the degenerated disks exhibited decreased volume and elasticity compared with those of normal disks (Fig. 1B). Histological characteristics were used to determine the extent of degeneration in each sample [20, 21]. Figure 1B shows that the IDD group had more severe degeneration than the NC group. Quantitative iTRAQ-based proteomic analysis of human intervertebral disk tissues revealed a significantly lower amount of DEPTOR in the NP tissues of degenerated disks than in the normal group (Fig. 1C). WB analysis of human NP tissues revealed significantly lower DEPTOR expression levels in degenerated disks than in normal disks (Fig. 1D). Immunofluorescence (IF) data from rat NP tissues revealed significantly lower DEPTOR expression in degenerated disks than in normal disks (Fig. 1E). These results, which combine MRI, histology, and quantitative proteomic analysis using iTRAQ, WB, and IF, show significantly decreased DEPTOR protein content in degenerated intervertebral disks.

### DOX induces NPC senescence while affecting mTORC1/S6K1/ATG1 expression

The degenerated intervertebral disks contained significantly more senescent cells. To investigate the alterations in DEPTOR and mTOR-related pathway proteins in senescent NPCs, an NPC senescence model was established, and DOX was used to induce NPC senescence. WB analysis showed that in senescent NPCs, mTORC1 and S6K1 levels were significantly upregulated, whereas DEPTOR and ATG1 expression levels were significantly downregulated (Fig. 1F, G). These findings imply that DEPTOR regulates NPC senescence via the mTORC1/S6K1/ATG1 pathway.

### DEPTOR can increase NPC viability and alleviate cell senescence

**Fig. 2:**
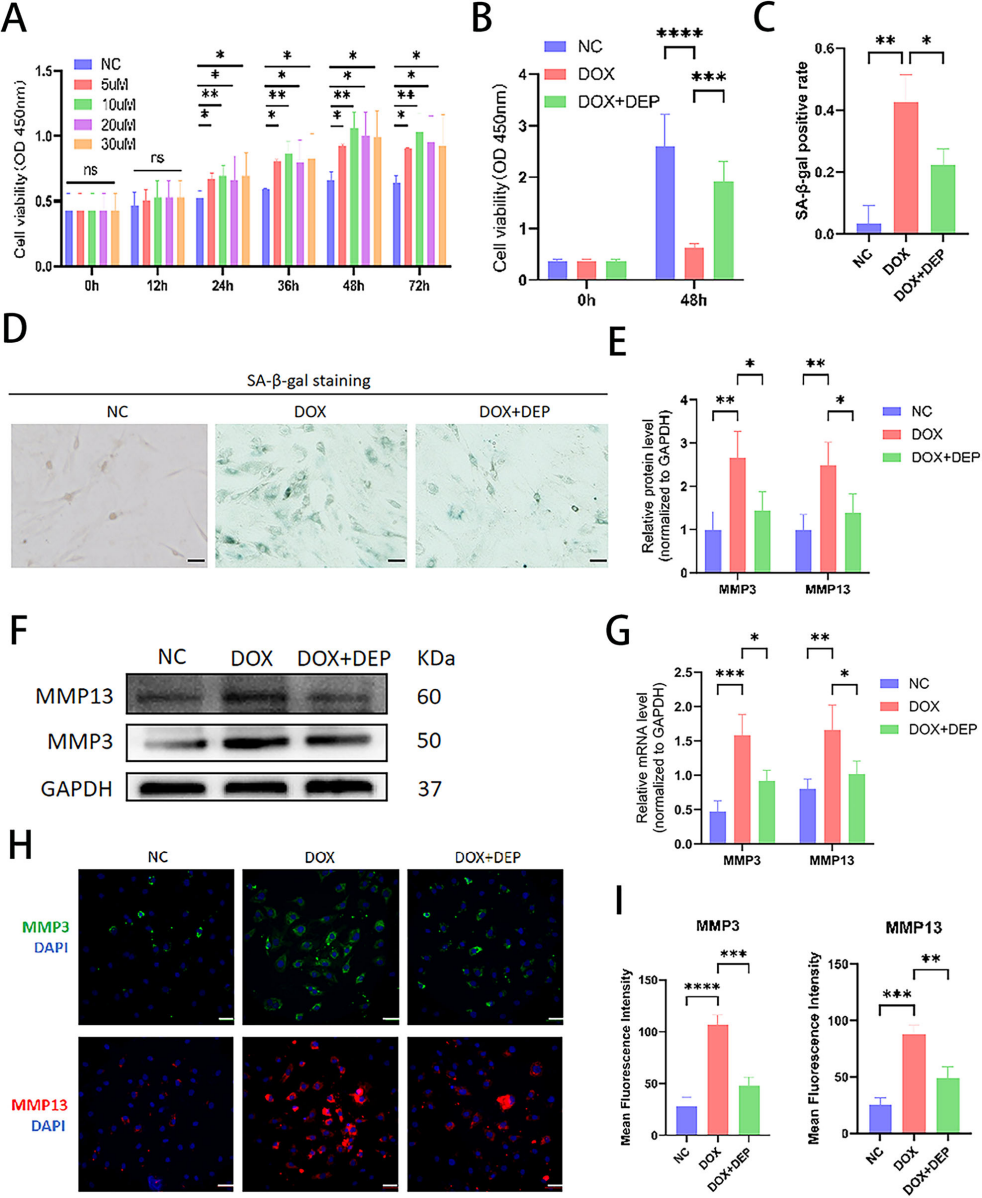
Effects of DEPTOR recombinant protein concentrations on NPC viability and metalloproteinase expression. **A** Viability of NPCs treated with various concentrations of DEPTOR recombinant protein. **B** Viability of DOX-induced senescent NPCs treated with DEPTOR recombinant protein. **C**, **D** β-galactosidase staining and quantitative analysis of NPCs; scale bar: 200 µm. **E**, **F** WB detection and quantitative analysis of metalloproteinases (MMP3 and MMP13) in DOX-induced senescent NPCs. **G** RT-PCR analysis of MMP3 and MMP13 in DOX-induced senescent NPCs. **H**, **I** Immunofluorescence analysis and quantification of metalloproteinases (MMP3 and MMP13) in DOX-induced NPCs; scale bar: 200 µm. Data are presented as means ± standard deviations; ns nonsignificant; *p < 0.05; **p < 0.01; ***p < 0.001; ****p < 0.0001.

To confirm whether DEPTOR could prevent NPC senescence, the senescent NPC model was used, and the recombinant DEPTOR protein was added to increase the DEPTOR levels. The CCK-8 analysis demonstrated that the DEPTOR recombinant protein increased NPC viability in a concentration-dependent manner, even at the lowest concentration (10 µM) (Fig. 2A). Although the DOX + DEPTOR recombinant protein group demonstrated a notable increase in NPC viability, the DOX-treated group showed significantly decreased NPC viability and increased cell senescence compared with those of the NC group (Fig. 2B). β-galactosidase (β-gal) staining revealed that the DEPTOR recombinant protein significantly inhibited NPC senescence (Fig. 2C, D). These findings imply that DEPTOR recombinant protein reduces NPC senescence and increases senescent NPC viability.

### DEPTOR promotes cellular autophagy via the mTORC1/ATG1 pathway and inhibits SASP secretion via the mTORC1/S6K1 pathway

**Fig. 3:**
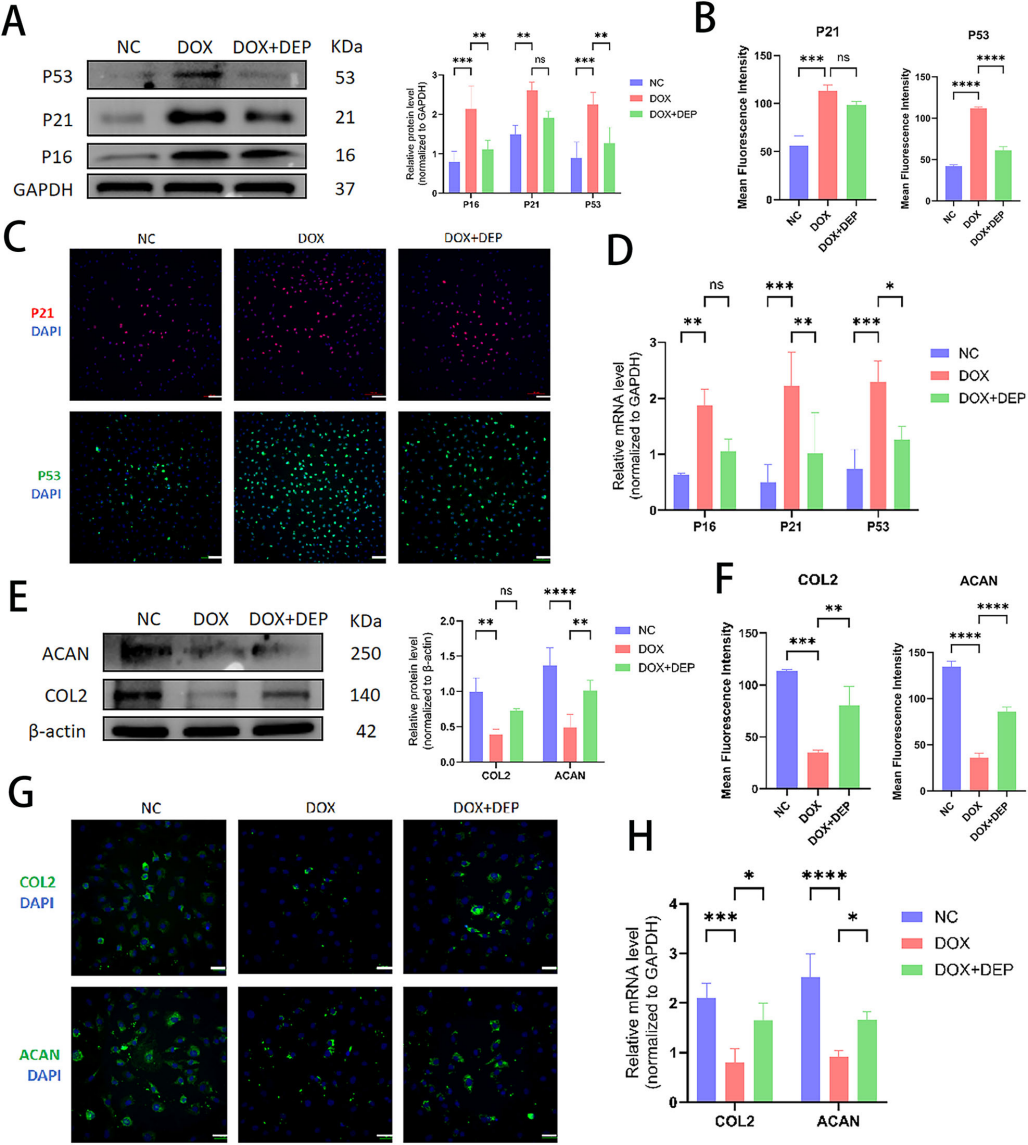
Role of DEPTOR in aging-related proteins and extracellular matrix regulation in NPCs. **A** WB detection and quantitative analysis of senescence markers P16, P21, and P53 in DOX-induced senescent NPCs. **B**, **C** Immunofluorescence and quantitative analysis of P16, P21, and P53 in DOX-induced NPCs; scale bar: 200 µm. **D** Reverse transcription–polymerase chain reaction (RT-PCR) analysis of P16, P21, and P53 expression in DOX-induced NPCs. **E** WB detection and quantitative analysis of extracellular matrix proteins (COL2 and ACAN) in DOX-induced NPCs. **F**, **G** Immunofluorescence and quantitative analysis of COL2 and ACAN in DOX-induced NPCs; scale bar: 200 µm. **H** RT-PCR analysis of COL2 and ACAN in DOX-induced NPCs. Results are presented as means ± standard deviations; ns nonsignificant; *p < 0.05; **p < 0.01; ***p < 0.001; ****p < 0.0001.

**Fig. 4:**
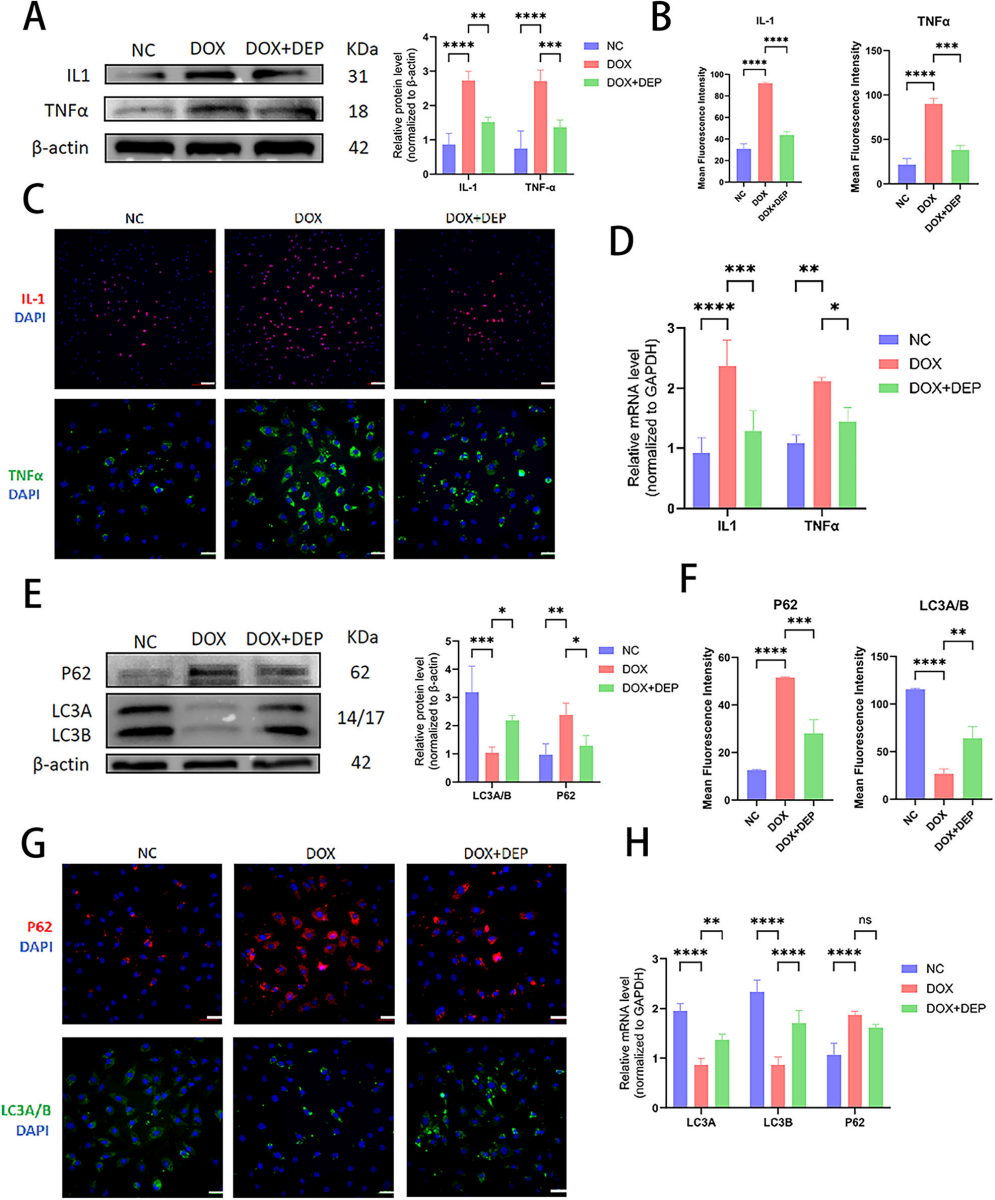
Role of DEPTOR in inflammation, chemokine regulation, and cellular autophagy in NPCs. **A** WB detection and quantitative analysis of DOX-induced NP inflammation and chemokines (IL-1 and TNFα). **B**, **C** Immunofluorescence and quantitative analysis of IL-1 and TNFα in DOX-induced NPCs; scale bar: 200 µm. **D** RT-PCR analysis of IL-1 and TNFα in DOX-induced NPCs. **E** WB detection and quantitative analysis of the autophagy markers LC3 A/B and P62 in DOX-induced NPCs. **F**, **G** Immunofluorescence and quantitative analysis of LC3 A/B and P62 in DOX-induced NPCs; scale bar: 200 µm. **H** RT-PCR analysis of LC3 A/B and P62 in DOX-induced NPCs. Data are presented as means ± standard deviations; ns nonsignificant; *p < 0.05; **p < 0.01; ***p < 0.001; ****p < 0.0001.

Building on earlier findings that senescent NPCs exhibit differential expression of proteins related to the mTOR pathway, we also investigated the mechanism by which DEPTOR regulates NPC senescence, specifically in connection with mTORC1/S6K1/ATG1. WB, PCR, and IF experiments showed consistent outcomes. In addition to the increased levels of aging-related proteins (P16, P21, and P53) (Fig. 3A–D), senescent NPCs showed increased synthesis of metalloproteinases (MMP3 and MMP13) (Fig. 2E–I), degradation of ECM components (COL2 and ACAN) (Fig. 3E–H), and elevated levels of inflammatory and chemotactic factors (IL-1 and TNFα) (Fig. 4A–D). Conversely, SASP expression was more prominent, and expression levels of cellular autophagy markers (LC3A/B and P62) decreased (Fig. 4E–H). Metalloproteinases (Fig. 2E–I), inflammatory cytokines and chemokines (Fig. 4A–D), and aging-related proteins (Fig. 3A–D) were all downregulated following addition of DEPTOR recombinant protein. Moreover, both ECM synthesis (Fig. 3E–H) and cellular autophagy (Fig. 4E–H) increased. In addition, a significant decrease was observed in SASP secretion. These results indicate that DEPTOR recombinant protein can inhibit SASP secretion and promote autophagy.

**Fig. 5:**
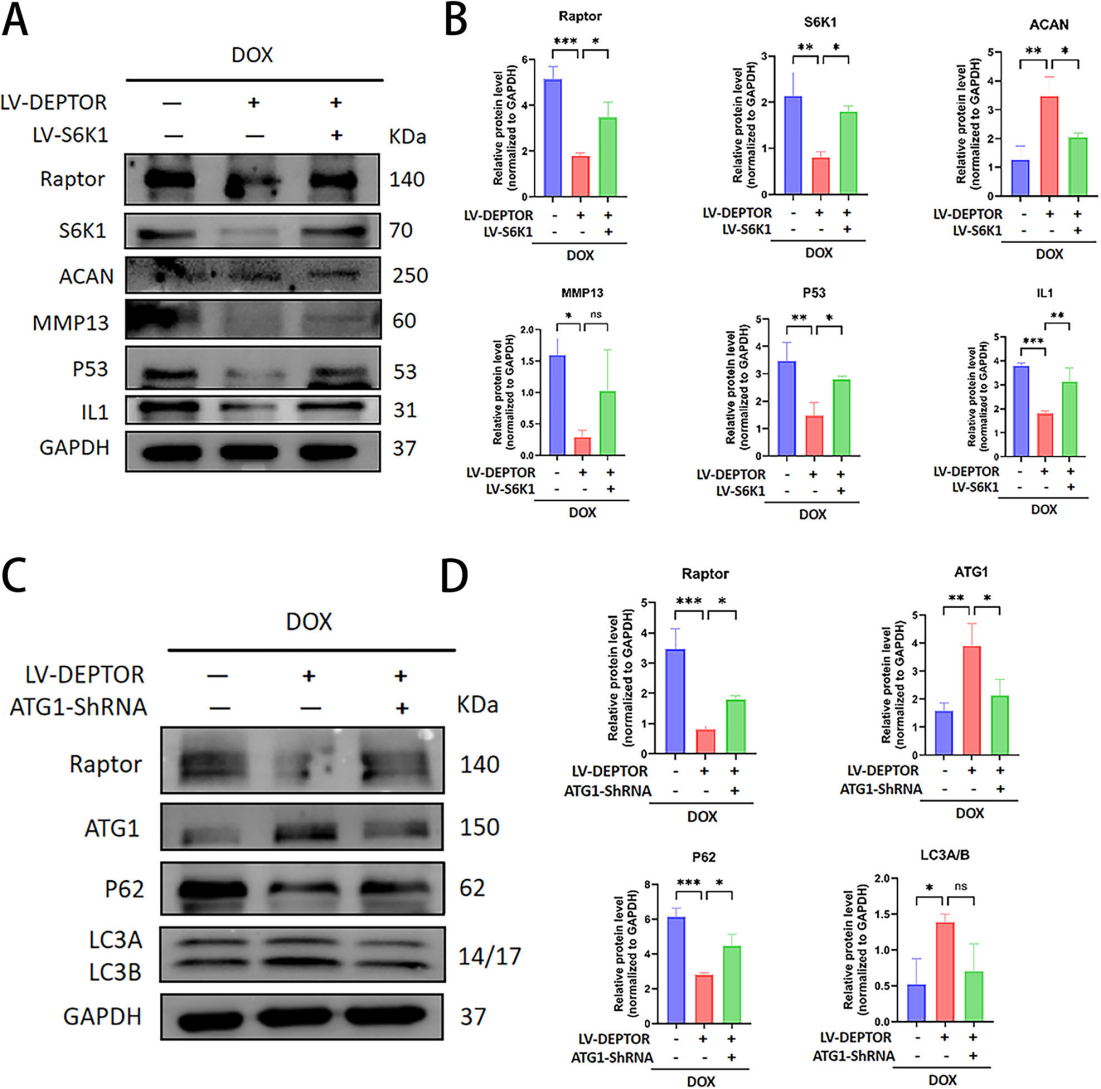
DEPTOR regulates autophagy and SASP in NPCs via S6K1 and ATG1 pathways. **A**, **B** WB detection and quantitative analysis of mTORC1, S6K1, ACAN, MMP13, P53, and IL-1 following DEPTOR and S6K1 overexpression in senescent NPCs. **C**, **D** WB detection and quantitative analysis of mTORC1, ATG1, P62, and LC3A/B following DEPTOR overexpression and ATG1 knockdown in senescent NPCs. Results are presented as means ± standard deviations; ns nonsignificant; *p < 0.05; **p < 0.01; ***p < 0.001; ****p < 0.0001.

Compared with the degeneration group, DEPTOR overexpression attenuated SASP and decreased the expression levels of mTORC1, S6K1, ACAN, MMP13, P53, and IL-1 in the mTORC1/S6K1 pathway. In DEPTOR-overexpressing NPCs, S6K1 upregulation was associated with increased secretion of SASP and expression of mTORC1, MMP13, P53, and IL-1 (Fig. 5A, B). This implies that S6K1 can effectively offset the inhibition of NPC senescence by DEPTOR. Compared with the degeneration group, DEPTOR overexpression increased cellular autophagy by downregulating mTORC1 and P62 and upregulating ATG1 and LC3A/B in the mTORC1/ATG1 pathway. Cellular autophagy decreased when ATG1 expression was decreased in DEPTOR-overexpressing NPCs; meanwhile, mTORC1 and P62 levels increased, whereas ATG1 and LC3A/B levels decreased (Fig. 5C, D). These results imply that ATG1 is essential for mediating the effect of DEPTOR on NPC senescence. Overall, these findings indicate that DEPTOR inhibits SASP via the mTORC1/S6K1 pathway and stimulates autophagy via the mTORC1/ATG1 pathway.

### DEPTOR alleviates IDD in rats

**Fig. 6:**
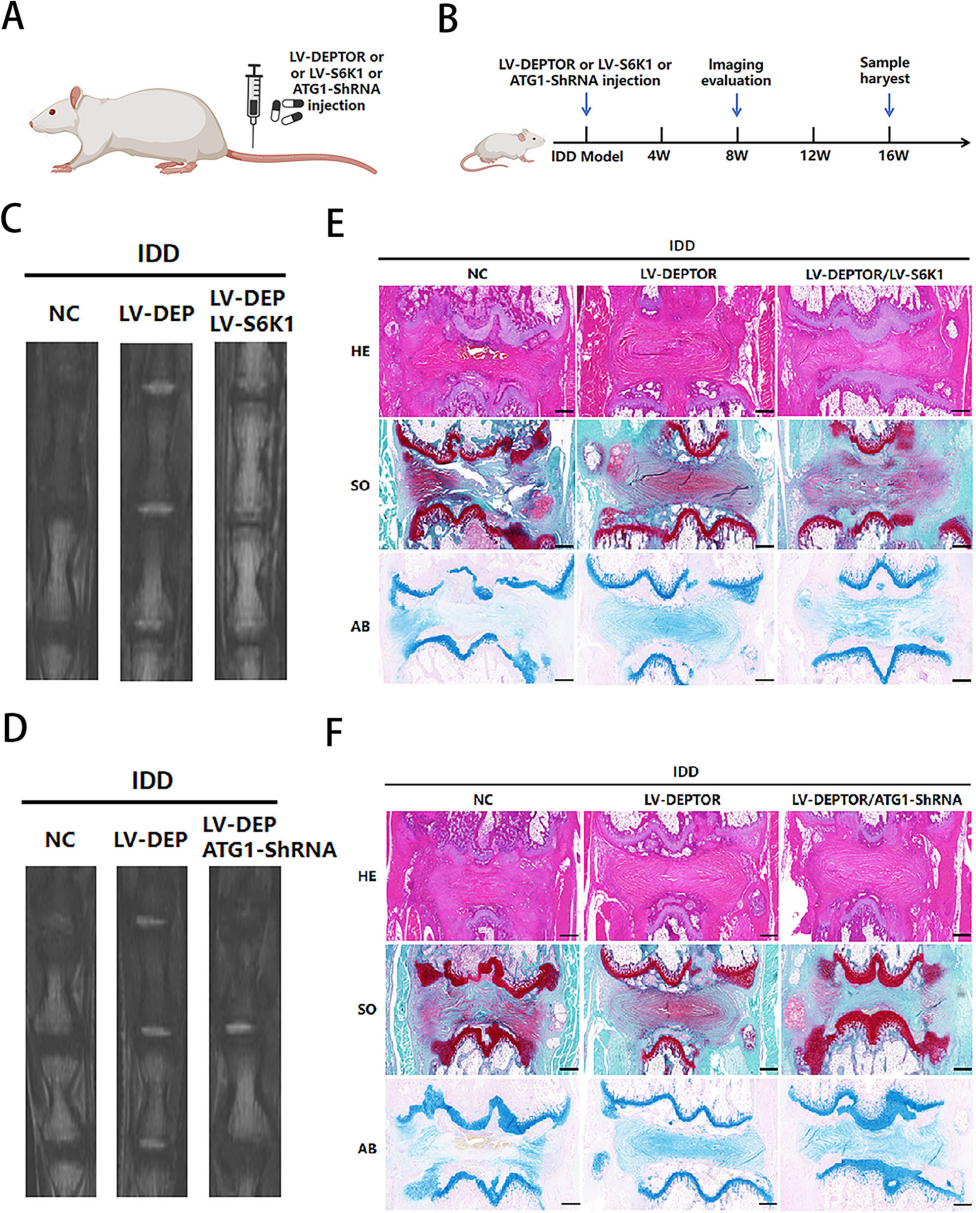
DEPTOR delays intervertebral disk degeneration (IDD) progression in a rat IDD model. **A**, **B** Overview and schematic of the study design and animal model (illustration generated using https://BioRender.com). **C**, **D** Magnetic resonance imaging (MRI) of caudal intervertebral disks. **E**, **F** Hematoxylin and eosin and Alcian blue staining of intervertebral disk sections; scale bar: 200 µm.

Our results were verified in vivo to further clarify the function and mechanism of DEPTOR in regulating NP aging (Fig. 6A, B).

**Fig. 7:**
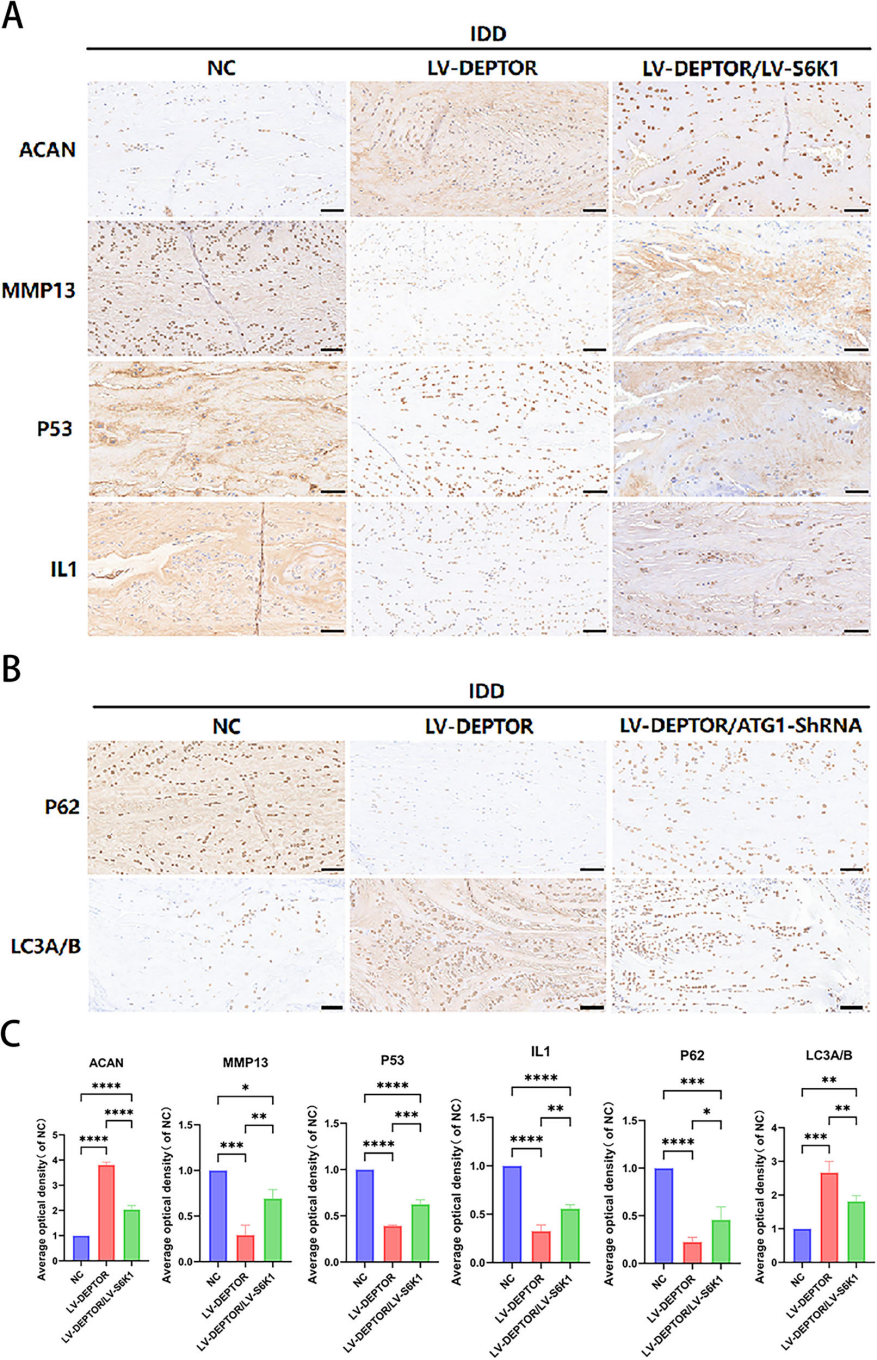
Immunohistochemical (IHC) staining and quantitative analysis of rat intervertebral disk sections. **A–C** IHC staining and quantification of target proteins in intervertebral disk sections; scale bar: 50 µm. Data are presented as means ± standard deviations; ns nonsignificant; *p < 0.05; **p < 0.01; ***p < 0.001; ****p < 0.0001.

T2WI revealed higher signal intensities in the intervertebral disks of the LV-DEPTOR group than in the degeneration group of the IDD rat model. After ATG1 downregulation (Fig. 6D) or S6K1 upregulation (Fig. 6C), the T2WI signal intensity decreased. Histological analysis revealed that the LV-DEPTOR group exhibited less disk tissue degeneration than the degeneration group (Fig. 6E), with increased ACAN expression (Fig. 7A and C) and decreased P53, MMP3, and TNFα levels, indicating decreased SASP. Conversely, S6K1 upregulation worsened disk degeneration (Fig. 6E); increased the expression levels of P53, MMP3, and TNFα; decreased the expression of ACAN (Fig. 7A and C); and increased SASP. Compared with the degeneration group, the LV-DEPTOR group showed lower severe disk degeneration (Fig. 6F), which was characterized by increased cellular autophagy, decreased P62 expression, and increased LC3A/B expression (Fig. 7B, C). Disk tissue degeneration that worsened following ATG1 downregulation led to P62 upregulation, LC3A/B downregulation (Fig. 7B, C), and cellular autophagy reduction (Fig. 6F). These findings imply that DEPTOR can inhibit SASP secretion via the mTORC1/S6K1 pathway and stimulate cellular autophagy via the mTORC1/ATG1 pathway. Histological and MRI evaluations together indicated that DEPTOR contributes to the delay of IDD progression in vivo.

## Discussion

The substantial medical and economic burden of degenerative disk–related disorders and LBP underscores the need for further investigation into their etiology and the development of efficient nonsurgical treatment strategies [22, 23]. Previous studies have shown that the degree of disk degeneration, as assessed by the Pfirrmann grading system, is positively correlated with the number of β-galactosidase-positive cells. In addition, the telomere length in these cells gradually decreases as the degree of degeneration intensifies, and the proliferation rate of NPCs isolated from degenerated disks is lower, whereas the senescence rate is substantially higher [24, 25]. Degenerated disks also exhibit the upregulation of the senescence-associated marker genes p16 and p53 along with the activation of the p16-Rb and p53-p21-Rb senescence pathways. The accumulation of senescent NPCs is a characteristic of disk degeneration [26, 27]. In this study, the IDD cell model exhibited an increase in senescent cells, whereas recombinant DEPTOR protein treatment decreased senescent cells. This intervention markedly increased both NPC senescence and the expression of proteins associated with aging.

Numerous disease models have demonstrated the tissue-specific and asymmetric effects of DEPTOR, a dual regulatory protein of mTORC1/2, on mTORC. DEPTOR regulates Akt phosphorylation and preserves metabolic homeostasis in skeletal muscle through mTORC2 [28]. However, it triggers autophagy and inhibits cell proliferation in the tumor microenvironment by blocking mTORC1 [29]. This functional diversity may be caused by the tissue-specific assembly pattern of the mTOR complex and its interaction with microenvironmental cues. DEPTOR inhibits mTORC1 in neurons to promote synaptic plasticity [30] and regulates lipid synthesis in adipocytes by inhibiting mTORC2 [31]. Furthermore, DEPTOR expression differs among tissues, and some studies have demonstrated that DEPTOR expression is significantly dependent on the tumor type. For instance, DEPTOR is highly expressed in prostate cancer and multiple myeloma and promotes tumor survival by activating the PI3K/Akt signaling pathway [29] but is less expressed in hepatocellular and colorectal cancers [28] and adipocytes [30]. However, in adipocytes, it promotes lipid synthesis via mTORC2 [31]. Decreased DEPTOR expression may deregulate mTORC2 inhibition, accelerating the epithelial–mesenchymal transition [32, 33]. DEPTOR modulates mTORC activity by both activating and inhibiting its functions. The tissue microenvironment, interplay between upstream and downstream signaling networks, or epigenetic changes (such as phosphorylation or ubiquitination regulation) may influence the biological effects of DEPTOR’s tissue-specific and asymmetric inhibitory influence on mTORC [18, 19]. This study confirmed the strong inhibitory effect of DEPTOR on mTORC1 and its significantly decreased expression in degenerating disk tissues.

Considering its “dual synergistic” and “precise regulatory” properties, DEPTOR offers distinct advantages over traditional mTOR inhibitors such as rapamycin. Although rapamycin effectively inhibits mTORC1, compensatory mTORC2 activation may have adverse effects, such as insulin resistance, after prolonged use [34]. DEPTOR, an endogenous regulator, maintains signaling homeostasis and reduces adverse effects by inhibiting mTORC1 activity while partially preserving mTORC2 activity [35].

The present study and our previous findings suggested a potential mechanistic connection between DEPTOR and IDD development, which revealed decreased DEPTOR expression in degenerated disks [13] and the effective inhibition of SASP secretion by exogenous DEPTOR recombinant protein. The present study revealed enhanced ECM degradation; increased inflammatory, chemotactic, and aging-related protein synthesis; and increased matrix-degrading enzyme (metalloproteinases) expression in the IDD cell model. These alterations were reversed by recombinant DEPTOR administration: ECM synthesis increased, whereas metalloproteinases, inflammatory cytokines, and aging-related proteins were downregulated. This finding indicates the effectiveness of DEPTOR supplementation in suppressing SASP.

DEPTOR overexpression resulted in decreased mTORC1 and S6K1 expression, decreased metalloproteinase levels, increased ECM production, and suppression of inflammatory and aging-related proteins, leading to decreased SASP secretion in both cellular and animal models of IDD. Conversely, S6K1 overexpression in the mTORC1/S6K1 pathway increased the expression of inflammatory mediators and matrix-degrading enzymes, reduced ECM synthesis, amplified SASP, and upregulated mTORC1 and S6K1 expression. These results imply that DEPTOR suppresses the mTORC1/S6K1 signaling axis, which in turn inhibits SASP in degenerated intervertebral disks.

The NF-κB and MAPK pathways regulate the expression of MMPs, which are key players in ECM breakdown (e.g., MMP-3/13) [36]. A study reported that SASP exacerbates ECM degradation through MMP secretion [37]. In the present study, the expression levels of MMPs (e.g., MMP-3/13) and metalloproteinases were significantly elevated in degenerated intervertebral disks, resulting in an imbalance in ECM catabolism. In addition to directly activating MMPs, an inflammatory microenvironment prevents collagen II and proteoglycan synthesis, which results in the structural degeneration of the intervertebral disk. By inhibiting the mTORC1/S6K1 axis, DEPTOR considerably reduces the amount of MMP-13, which is consistent with the findings in the osteoarthritis model [38].

The key elements of SASP include proinflammatory cytokines such as TNFα and IL-1. In this study, their expression was significantly increased in degenerated disks, where they propagated inflammatory responses via paracrine mechanisms. These cytokines are reported to regulate the NF-κB pathway to induce the senescence of nearby cells [39]. Our findings provide direct experimental evidence supporting the theory that SASP induces disk inflammation, demonstrating that DEPTOR treatment suppressed cytokine release and attenuated the inflammatory cascade.

Another feature of disk degeneration is the accumulation of proteins associated with aging [40]. In the degenerated disks, P16, P21, and P53 expression were increased, which correlated with SASP activation. The concept that targeting senescent cells can alleviate IDD is supported by the finding that DEPTOR treatment reduces the expression of these proteins, demonstrating its function in reducing cellular senescence by modulating senescence signaling pathways [41].

Autophagic efficiency is reported to decrease with age, and DEPTOR stimulates autophagy by directly inhibiting mTORC1 independent of Akt [42]. By phosphorylating ATG1 and blocking the ULK1/Atg13/FIP200 complex, mTORC1 inhibited autophagy and prevented autophagosome formation. In the rat and IDD cell models, DEPTOR overexpression increased cellular autophagy, decreased mTORC1 expression, and increased ATG1 expression compared with the degeneration group. After ATG1 downregulation, cellular autophagy was reduced, mTORC1 expression was upregulated, and ATG1 expression was downregulated. This implies that DEPTOR inhibits SASP secretion via the mTORC1/ATG1 pathway and stimulates cellular autophagy in degenerated intervertebral disks.

## Conclusions

**Fig. 8:**
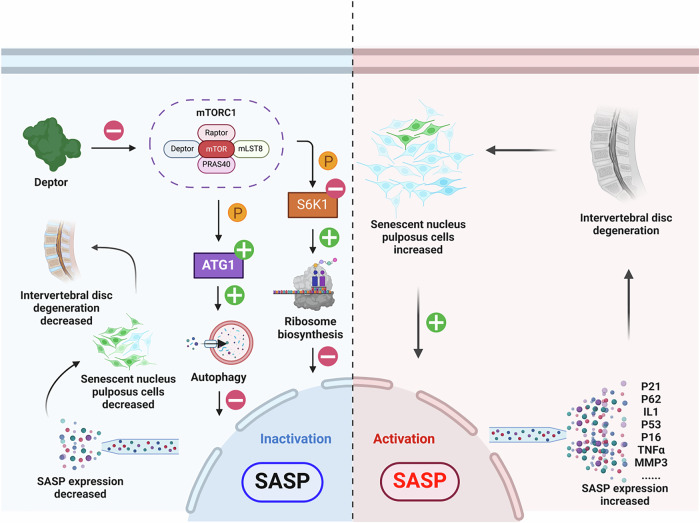
Schematic diagram summarizing the study. DEPTOR promotes cellular autophagy via the mTORC1/ATG1 pathway and together with the mTORC1/S6K1 pathway suppresses SASP secretion to alleviate IDD (illustration generated using https://BioRender.com).

The pathophysiology of IDD is intricately linked to decreased DEPTOR expression. The in vitro and in vivo results of this study show that DEPTOR alleviates IDD by suppressing SASP secretion by inhibiting the mTORC1/S6K1 pathway and promoting cellular autophagy via the mTORC1/ATG1 pathway. These findings provide encouraging avenues for developing therapeutic targets for degenerative disk disease and demonstrate the potential of DEPTOR as a novel cytokine involved in NPC regulation (Fig. 8).

## Materials and methods

### Acquisition of human and rat tissue specimens

NP tissues were collected from patients undergoing spinal surgery at The Affiliated Hospital of Qingdao University. Degenerative NP tissues were obtained from patients with disk degeneration, whereas normal human lumbar spine NP tissues were collected from patients with idiopathic scoliosis but without IDD [43]. All patients were free of diabetes, liver or kidney disease, tumors, immune disorders, and infections. Three senior spine surgeons and one senior imaging surgeon evaluated the patients via magnetic resonance imaging (MRI) before surgery. Disk degeneration was graded using the Pfirrmann classification [44]. All procedures were approved by the Ethics Committee of The Qingdao University Affiliated Hospital.

Animal experiments were conducted in accordance with the guidelines of the Experimental Committee of Qingdao University Affiliated Hospital. In total, 24 male Sprague Dawley (SD) rats (8 weeks old) were purchased from Beijing Vital River Laboratory Animal Co., Ltd. and housed under a 12/12-h light/dark cycle at 20 °C–25 °C and 40%–60% humidity for a 1-week acclimation period. Rats were randomly assigned to the control or experimental groups via simple randomization, with investigators and data analysts blinded to group assignments. The experimental procedures strictly followed the Regulations on the Management of Laboratory Animals [45].

### Isolation, culture, and senescent cell modeling of NPCs

Primary rat NP tissues were isolated following a published protocol [46]. Briefly, NP tissues from five SD rat lumbar intervertebral disks were digested using type II collagenase for 30 min at 37 °C. After filtering through a cell strainer, digested tissues were cultured in 25-cm^2^ flasks, and microtissues were discarded. The culture process was as follows [47]: cells were seeded at a density of 1 × 10^5^ cells/mL in DMEM/F12 medium containing 10% fetal bovine serum and incubated at 37 °C with 5% CO_2_. Upon reaching 80%–90% confluence, cells were trypsinized and subcultured at 37 °C. After three passages, homogeneous spindle-shaped cells were observed. Subsequent experiments used NPCs from passages 3–5. Human-derived NPCs were cultured under similar conditions. To induce senescence, NPCs in the logarithmic phase were treated with 100-nM doxorubicin (DOX) for 48 h.

### Construction and transfection of lentiviral vector

NPCs were stably transfected with LV-DEPTOR or LV-NC according to the relevant protocol. Western blotting (WB) confirmed DEPTOR overexpression, indicating increased DEPTOR protein expression in NPCs. WB was also used to detect ATG1 and S6K1 protein levels after similar techniques were employed to establish knockdown with ATG1-ShRNA and overexpress S6K1 via LV-S6K1, respectively, in NPCs. ATG1-ShRNA, LV-DEPTOR, and LV-S6K1 were procured from GeneChem.

### Isobaric tags for relative and absolute quantitation

Proteins from healthy and degenerated human intervertebral disk tissues were extracted, reduced, alkylated, and digested with trypsin. Peptides were labeled independently using isotope-labeled isobaric tags for relative and absolute quantitation (iTRAQ) reagents. To reduce sample complexity, the labeled samples were pooled for separation via high-performance liquid chromatography (HPLC). Peptides were analyzed via HPLC-tandem mass spectrometry. Reporter ions from isotopically labeled peptides enabled the relative quantification of peptides across samples. Mass spectrometry data were processed for quantitative and statistical analysis, and differentially expressed proteins were identified using the bioinformatics software Proteome Discoverer. Functional annotations were used to assess their biological significance.

### Conventional WB analysis

Total protein was extracted using RIPA lysis buffer, and protein concentrations were measured using the bicinchoninic acid analysis. Proteins were mixed with 5× protein loading buffer (1:4 ratio), denatured for 10 min at 95 °C, cooled, and stored at −20 °C. Samples were resolved using sodium dodecyl sulfate–polyacrylamide gel electrophoresis, transferred to polyvinylidene difluoride membranes, and blocked in 5% skim milk for 2 h. The membranes were incubated overnight with primary antibodies, followed by horseradish peroxidase–conjugated secondary antibodies for 1 h at room temperature. Gray values were measured using image analysis software, and proteins were detected using the enhanced chemiluminescence method. Details of the antibodies used are provided in Table 1.

**Table 1:** Antibody information.

Antibody name	Product number	Antibody name	Product number
DEPTOR	ab244395	P53	ab32049
MMP3	17873-1-AP	ACAN	68350-1-Ig
MMP13	18165-1-AP	COL2	28459-1-AP
IL-1beta	16806-1-AP	LC3	81004-1-RR
TNF-alpha	60291-1-Ig	P62	18420-1-AP
P16-INK4A	10883-1-AP	Beta actin	20536-1-AP
P21	ab109520	GAPDH	10494-1-AP

The table includes antibody name and product number.

### Conventional reverse transcription–polymerase chain reaction analysis

Total RNA was extracted using the Trizol method, and its quality and concentration were assessed by measuring optical density at 260 and 280 nm. RNA was used as a template for two-step reverse transcription to generate single-stranded DNA. Forward and reverse primers were designed for DEPTOR, matrix metalloproteinases (MMP3 and MMP13), inflammatory cytokines and chemokines (IL-1 and TNFα), aging-related proteins (P16, P21, and P53), extracellular matrix proteins (COL2 and ACAN), microtubule-associated proteins (LC3A/B), and autophagy-related proteins (P62). β-actin served as the internal control for fluorescence quantitative polymerase chain reaction (PCR), which was performed using a PCR kit. Each sample was tested in triplicate, and the means were calculated. The relative mRNA expression was analyzed using the 2^−ΔΔCt^ method.

### Immunofluorescence

Tissue or cell samples were fixed with paraformaldehyde. After fixation, a permeabilizing agent, such as Triton ×-100, was applied to aid antibody penetration. Nonspecific binding sites were blocked using a sealing agent, such as bovine serum albumin. Primary antibodies were then added and incubated to bind the target protein. After washing, the samples were incubated with a fluorescently labeled secondary antibody. After additional washes, the samples were sealed and imaged with a fluorescence microscope. Comprehensive antibody details are listed in Table 1.

### Establishment of IDD model rats

Rats were acclimated for 1 week before modeling. The day before the procedure, the rats were deprived of food and water. The IDD model was established using the needle puncture method [48]. After intraperitoneal injection of pentobarbital to induce anesthesia, the first caudal vertebra was identified by palpating the Co5–Co6, Co6–Co7, and Co7–Co8 disks from the tail root downward. The caudal disk was punctured using a syringe needle, inserted 5 mm through the annulus fibrosus into the NP and rotated 360°. A portion of NP tissue was aspirated with an empty 5-mL needle before withdrawal. After 8 weeks, IDD modeling was deemed successful, and LV-DEPTOR, ATG1-ShRNA, and LV-S6K1 were injected into the Co5–Co6, Co6–Co7, and Co7–Co8 disks.

### Morphological observation and histological testing of intervertebral disks

After 8 weeks, MRI scans were performed using a 7.0 T small-animal MRI scanner following LV-DEPTOR, ATG1-ShRNA, and LV-S6K1 injections into the Co5–Co6, Co6–Co7, and Co7–Co8 disks in the rat IDD model. After scanning, the disks were decalcified, embedded in paraffin, sectioned, and stained.

Hematoxylin and Eosin Staining: Paraformaldehyde-fixed NP tissue was dehydrated, embedded in paraffin, and sectioned at a thickness of 5 μm. Sections were oven-dried, rinsed, dewaxed, stained with hematoxylin, dehydrated, and cleared. Slides were mounted with neutral resin and imaged using a light microscope.

Safranin O-Solid Green Staining: After deparaffinization, the sections were stained with freshly prepared Weigert solution for 3–5 min, rinsed, differentiated in acidic solution for 15 s, washed in distilled water for 10 min, stained with solid green for 5 min, washed in weak acid for 5 min, counterstained with safranin O for 5 min, dehydrated in 95% and absolute ethanol, cleared in xylene, and mounted with neutral resin.

Alcian Blue Staining: Dewaxed sections were stained with Alcian blue solution for 30 min, rinsed with distilled water, and counterstained with nuclear fast red for 5 min. After dehydration, clearing, and mounting, the sections were examined for blue precipitates of acidic mucopolysaccharides under a microscope.

Immunohistochemical Staining: Target proteins were stained via immunohistochemical (IHC) staining. After antigen retrieval, paraffin sections were dewaxed to water, incubated with 3% H_2_O_2_ for 10 min at room temperature, washed twice with phosphate-buffered saline (PBS) for 5 min each, and blocked with 5% goat serum in PBS for 1 h. Sections were then incubated with corresponding primary antibodies overnight at 4 °C, rinsed three times in PBS for 5 min per wash, and incubated with horseradish peroxidase–labeled secondary antibodies for 30 min at 37 °C. After three 5-min washes with PBS, DAB was applied for 3–15 min for color development. Sections were rinsed, counterstained, dehydrated, cleared with xylene, and mounted with neutral resin. Details of the antibodies are provided in Table 1.

### Statistical analysis

Unless otherwise specified, all data are presented as means ± standard deviations. GraphPad Prism (GraphPad Software Inc., CA, USA) was used to analyze data from at least three independent experiments. Differences between two groups were evaluated using Student’s *t*-test. One-way ANOVA followed by Tukey’s post hoc test was used for multiple group comparisons. Statistical significance was set at *p* < 0.05.

### Ethics approval and consent to participate

All human samples were obtained after obtaining the consent of the patients and their families through written informed consent forms. The study adhered to all ethical guidelines and regulations for the care and use of laboratory human samples, and all procedures were carried out in accordance with the approved protocol.

## Supplementary information

Supplementary results

Full and uncropped western blots

## Data Availability

RNA sequencing data are deposited in the Gene Expression Omnibus (GEO) datasets (https://www.ncbi.nlm.nih.gov/geo/).
