# Supplementary material for: DEPTOR regulates nucleus pulposus cell senescence through the mTORC1/S6K1/ATG1 pathway to alleviate intervertebral disk degeneration
Source: Cell Death Discov. 2025 Nov 17;11:533. doi: 10.1038/s41420-025-02819-9 (PMC12624031; doi:10.1038/s41420-025-02819-9)
Supplement: Supplementary file 1 — Supplementary results [file 41420_2025_2819_MOESM1_ESM.docx]

**Supplementary results**

**Materials and Methods**

***Conventional RT-PCR analysis***

Each sample group's total RNA was extracted using the Trizol method, and the quality and concentration of the RNA were evaluated by measuring the OD values at 260 and 280 nm. The RNA was used as a template for a two-step reverse transcription process that created single-stranded DNA. For the following target genes, forward and reverse primer sequences were created: DEPTOR; matrix metalloproteinases (MMP3, MMP13); inflammatory cytokines and chemokines, IL-1, and TNFα; cell cycle-associated proteins, P16, P21, and P53; extracellular matrix proteins, COL2, and ACAN; microtubule-associated protein LC3A/B; and autophagy-related proteins, P62. β-actin was used as the internal reference in fluorescence quantitative PCR, which was carried out using a PCR kit. For every sample, three replicate wells were used, and the average was determined. The 2^-ΔΔCt^ method was used to calculate the relative expression of each gene based on relative mRNA expression. The primers used are shown in **Table S1**.

***Antibody-related information***

The Antibody-related information is shown in **Table S2**.

**Results**

**Explanation of Figure 2A**

Figure 2A demonstrates that, compared with the NC group, all four concentrations of recombinant DEPTOR protein (5 µM, 10 µM, 20 µM, 30 µM) enhance the NPC viability, with the 10 µM concentration producing a significantly greater effect than the others. From the perspective of time, across all DEPTOR concentrations (NC, 5 µM, 10 µM, 20 µM, and 30 µM), no significant differences in cell viability were observed at 0 and 12 h. However, at 24, 36, 48, and 72h, cell viability increased significantly, peaking at 48 h. Therefore, this study used 10 µM recombinant DEPTOR protein with a 48 h incubation period for NP cell observations and subsequent experiments, which is reflected in Figure 2B. To support these results, the data for Figure 2A and its explanation have been included in the supplementary materials. The concentrations of DEPTOR recombinant protein and observation period are shown in **Table S3**.

**Discussion**

Autophagy removes damaged organelles and protein aggregates, which underscores its importance in preserving intracellular homeostasis. Long-term SASP activation has been associated with autophagy defects [1]. Consistent with the findings of Livingston et al. [2], the results of the present showed that DEPTOR decreased the accumulation of senescence markers (e.g., P16 and P53) and increased autophagy via the mTORC1/ATG1 axis in senescent fibroblasts. In addition, autophagy activation indirectly reduced the secretion of TNFα and IL-1β, indicating that autophagy may alleviate IDD progression by breaking the SASP-mediated positive feedback loop.

The proanabolic factor S6K1, a downstream effector of mTORC1, is implicated in matrix degradation and inflammation [3]. In line with the findings of Chen et al. in their study of chondrocytes [4], our S6K1 overexpression experiments verified that the inhibitory effects of DEPTOR on SASP are dependent on the suppression of the mTORC1/S6K1 axis. Our findings support the notion that S6K1 increases ribosomal biosynthesis, which exacerbates senescence [5]. This finding further clarifies how DEPTOR reduces SASP via the mTORC1/S6K1 pathway. Furthermore, mTORC1 directly inhibits the activity of ATG1, a kinase crucial in the initiation of autophagy [6]. Similar to the mechanisms observed in hepatocellular carcinoma cells, this study showed that DEPTOR promotes autophagosome formation by de-repressing ATG1 through mTORC1 inhibition [7]. These findings strengthened the conceptual framework of DEPTOR-mediated dual regulation through the mTORC1/S6K1 and mTORC1/ATG1 pathways.

**References:**

[1]Kang C, Xu Q, Martin TD, et al. The DNA damage response induces inflammation and senescence by inhibiting autophagy of GATA4[J].Science. 2015 Sep 25;349(6255):aaa5612.

[2]Livingston MJ, Shu S, Fan Y, et al. Tubular cells produce FGF2 via autophagy after acute kidney injury leading to fibroblast activation and renal fibrosis[J].Autophagy. 2023 Jan;19(1):256-277.

[3]Castillo Díaz F, Mottarlini F, Targa G, et al. Recency memory is altered in cocaine-withdrawn adolescent rats: Implication of cortical mTOR signaling[J].Prog Neuropsychopharmacol Biol Psychiatry. 2023 Dec 20;127:110822.

[4]Ji X, Ito A, Nakahata A, et al. Effects of in vivo cyclic compressive loading on the distribution of local Col2 and superficial lubricin in rat knee cartilage[J].J Orthop Res. 2021 Mar;39(3):543-552.

[5]Laplante M, Sabatini DM. mTOR signaling in growth control and disease[J].Cell. 2012 Apr 13;149(2):274-93.

[6]Ma L, Li K, Guo Y, et al. Selenium triggers AMPK-mTOR pathway to modulate autophagy related to oxidative stress of sheep Leydig cells[J].Reprod Biol. 2025 Mar;25(1):100973.

[7]Chan YC, Chang YC, Chuang HH, et al. Overexpression of PSAT1 promotes metastasis of lung adenocarcinoma by suppressing the IRF1-IFNγ axis[J].Oncogene. 2020 Mar;39(12):2509-2522.

**Table S1**. Primer sequences used for PCR

| Gene | Species | Direction | Sequences 5’-3’ |
| --- | --- | --- | --- |
| MMP3 | Rat | Forward | 5' ATGAAGAGTCTTCCAATCCTACTGT 3' |
|  |  | Reverse | 5' ACAGTAGGATTGGAAGACTCTTCAT 3' |
| MMP13 | Rat | Forward | 5' ATGCATCCAGGGGTCCTGGCTGCCT 3' |
|  |  | Reverse | 5' AGGCAGCCAGGACCCCTGGATGCAT 3' |
| IL-1beta | Rat | Forward | 5' TGTGATGTTCCCATTAGAC 3' |
|  |  | Reverse | 5' AATACCACTTGTTGGCTTA 3' |
| TNF-alpha | Rat | Forward | 5' CCACGCTCTTCTGTCTACTG 3' |
|  |  | Reverse | 5' GCTACGGGCTTGTCACTC 3' |
| P16-INK4A | Rat | Forward | 5' GGGAGGGCTTCCTAGACACT 3' |
|  |  | Reverse | 5' CTTGAGCAGAAGTTATGCCTGT 3' |
| P21 | Rat | Forward | 5' AGTAGACACGAAACAGGC 3' |
|  |  | Reverse | 5' TTCCCATCTTTGCTCATC 3' |
| P53 | Rat | Forward | 5' ATGAGTCTTCACAAGTCCGCGTCGT 3' |
|  |  | Reverse | 5' ACGACGCGGACTTGTGAAGACTCAT 3' |
| ACAN | Rat | Forward | 5' TCTACCCAGCACCCTACA 3' |
|  |  | Reverse | 5' GGAAAGTGGCGATAACAG 3' |
| COL2 | Rat | Forward | 5' AGAGCGGAGACTACTGGATTG 3' |
|  |  | Reverse | 5' TCTGGACGTTAGCGGTGTT 3' |
| LC3A | Rat | Forward | 5' TTCGCCGACCGCTGTAAG 3' |
|  |  | Reverse | 5' ATCCGTCTTCATCCTTCTCCTG 3' |
| LC3B | Rat | Forward | 5' TCCTGGACAAGACCAAGTT 3' |
|  |  | Reverse | 5' TCTCCTGGGAGGCATAGA 3' |
| P62 | Rat | Forward | 5' TGACGACTGGACGCATTT 3' |
|  |  | Reverse | 5' GTCTGTAGGAGCCTGGTGAG 3' |

**Table S2** Antibody-related information

| Antibody  Name | Product  Number | WB | IF | IHC | Company Identification |
| --- | --- | --- | --- | --- | --- |
| DEPTOR | ab244395 | 1:1000 | 1:200 | 1:200 | Proteintech |
| MMP3 | 17873-1-AP | 1:1000 | 1:200 | 1:100 | Proteintech |
| MMP13 | 18165-1-AP | 1:1000 | 1:200 | 1:200 | Proteintech |
| IL-1beta | 16806-1-AP | 1:5000 | 1:200 | 1:200 | Proteintech |
| TNF-alpha | 60291-1-Ig | 1:2000 | 1:200 | 1:200 | Proteintech |
| P16-INK4A | 10883-1-AP | 1:2000 | 1:400 | 1:2000 | Proteintech |
| P21 | ab109520 | 1:1000 | 1:100 | 1:200 | Abcam |
| P53 | ab32049 | 1:1000 | 1:100 | 1:50 | Abcam |
| ACAN | 68350-1-Ig | 1:1000 | 1:200 | 1:200 | Proteintech |
| COL2 | 28459-1-AP | 1:1000 | 1:200 | 1:200 | Proteintech |
| LC3 | 81004-1-RR | 1:5000 | 1:1000 | 1:500 | Proteintech |
| P62 | 18420-1-AP | 1:5000 | 1:1000 | 1:400 | Proteintech |
| Beta actin | 20536-1-AP | 1:5000 | 1:400 | 1:400 | Proteintech |
| GAPDH | 10494-1-AP | 1:5000 | 1:1000 | 1:400 | Proteintech |

**Table S3**. The effects of different concentrations of DEPTOR recombinant protein

on the viability of NP cells

| Concentration | Observation period | | | | | |
| --- | --- | --- | --- | --- | --- | --- |
|  | 0h | 12h | 24h | 36h | 48h | 72h |
| NC | 0.5688 | 0.5788 | 0.5882 | 0.5960 | 0.6147 | 0.6566 |
|  | 0.3994 | 0.4494 | 0.4742 | 0.5940 | 0.6194 | 0.6178 |
|  | 0.3177 | 0.3772 | 0.4970 | 0.5794 | 0.7358 | 0.6555 |
| 5µM | 0.5688 | 0.5882 | 0.6899 | 0.7780 | 0.9234 | 0.9020 |
|  | 0.3994 | 0.4994 | 0.7054 | 0.8056 | 0.9120 | 0.9091 |
|  | 0.3177 | 0.4177 | 0.6100 | 0.8237 | 0.9345 | 0.9088 |
| 10µM | 0.5688 | 0.5368 | 0.7088 | 0.9189 | 1.1905 | 1.1865 |
|  | 0.3994 | 0.5311 | 0.6990 | 0.8500 | 0.9964 | 0.9489 |
|  | 0.3177 | 0.5180 | 0.6781 | 0.8170 | 0.9998 | 0.9663 |
| 20µM | 0.5688 | 0.5281 | 0.6618 | 0.7696 | 1.0005 | 0.9578 |
|  | 0.3994 | 0.5205 | 0.6986 | 0.7993 | 0.9945 | 0.9544 |
|  | 0.3177 | 0.5372 | 0.6255 | 0.8170 | 1.0065 | 0.9613 |
| 30µM | 0.5688 | 0.5558 | 0.7081 | 0.7268 | 0.9872 | 0.9509 |
|  | 0.3994 | 0.5001 | 0.6899 | 0.8934 | 0.9814 | 0.9140 |
|  | 0.3177 | 0.5300 | 0.6879 | 0.8157 | 0.9931 | 0.9068 |
