# Supplementary material for: DEPTOR regulates nucleus pulposus cell senescence through the mTORC1/S6K1/ATG1 pathway to alleviate intervertebral disk degeneration
Source: Cell Death Discov. 2025 Nov 17;11:533. doi: 10.1038/s41420-025-02819-9 (PMC12624031; doi:10.1038/s41420-025-02819-9)
Supplement: Supplementary file 2 — Full and uncropped western blots [file 41420_2025_2819_MOESM2_ESM.docx]

**Full and uncropped western blots**

**Uncropped gels for Western Blots in Figure 1**

**Figure 1D of WB**


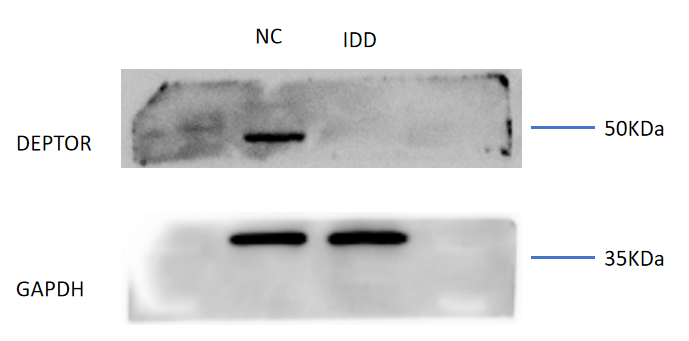


**Figure 1F of WB**

**
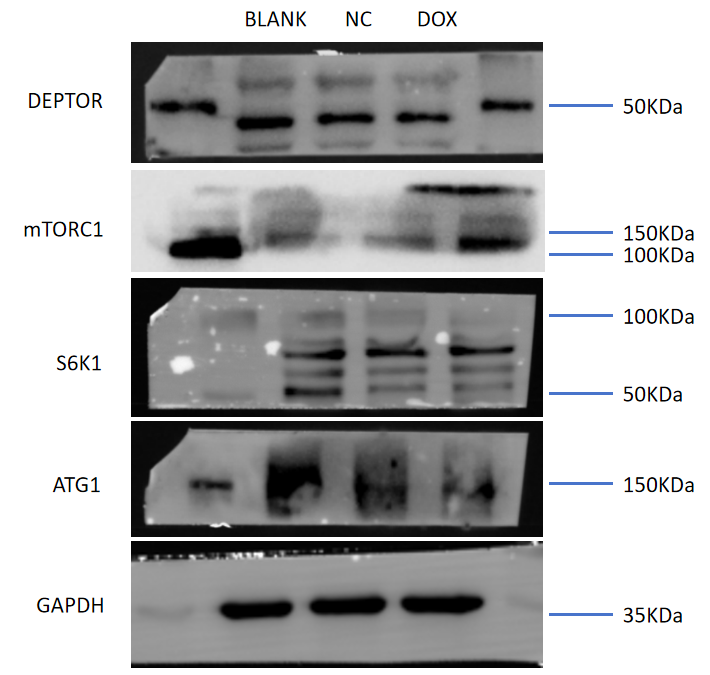
**

**Uncropped gels for Western Blots in Figure 2**

**Figure 2F of WB**

**
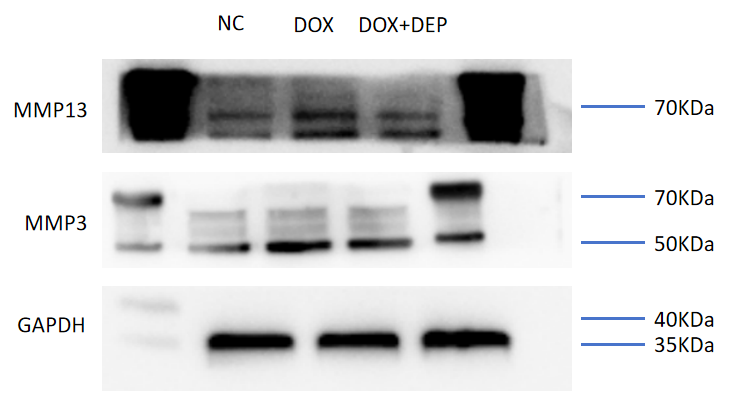
**

**Uncropped gels for Western Blots in Figure 3**

**Figure 3A of WB**

**
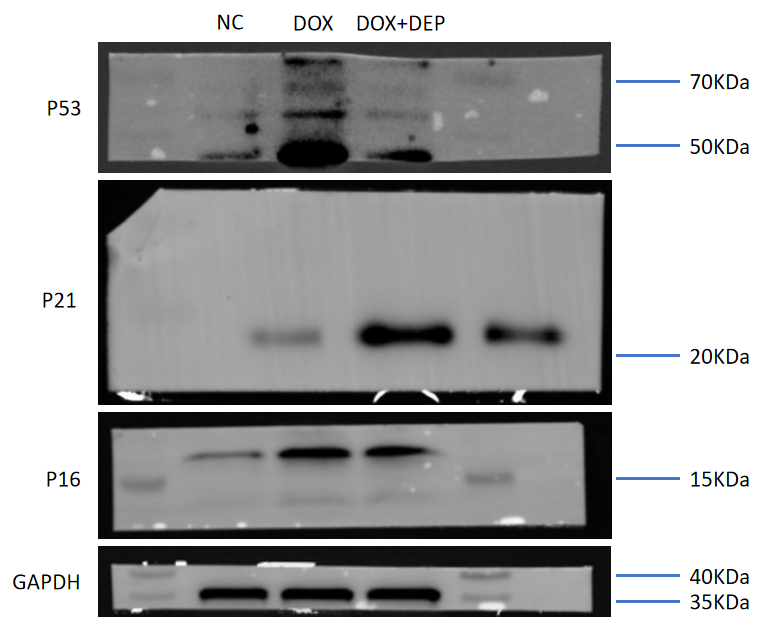
**

**Figure 3E of WB**

**
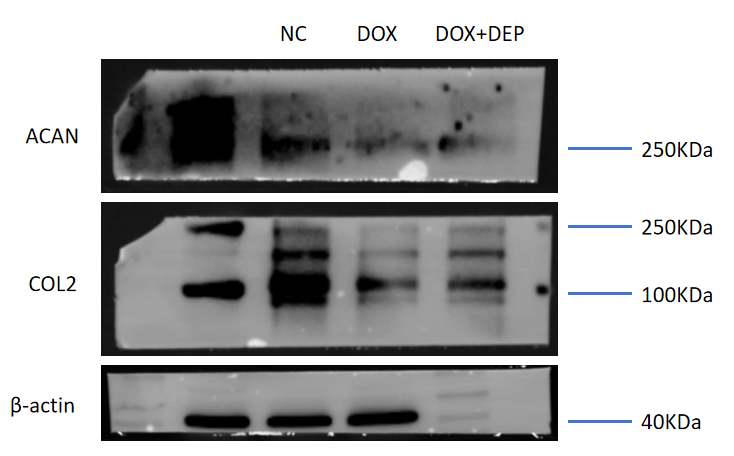
**

**Uncropped gels for Western Blots in Figure 4**

**Figure 4A of WB**

**
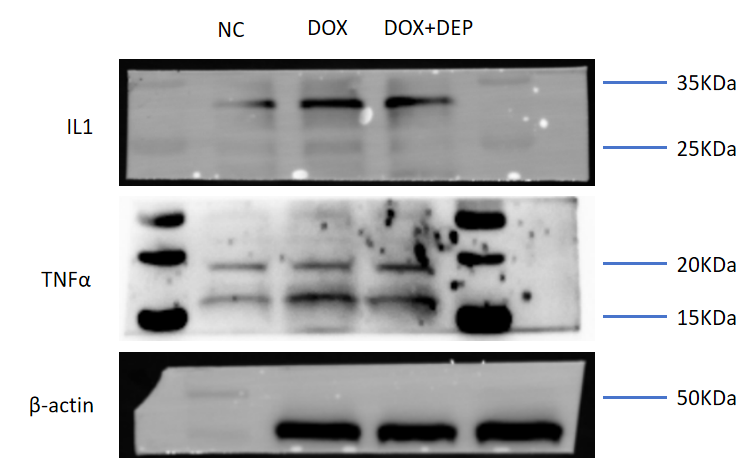
**

**Figure 4E of WB**

**
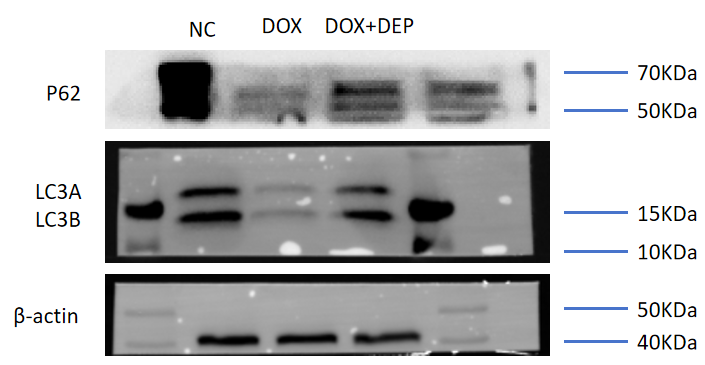
**

**Uncropped gels for Western Blots in Figure 5**

**Figure 5A of WB**

**
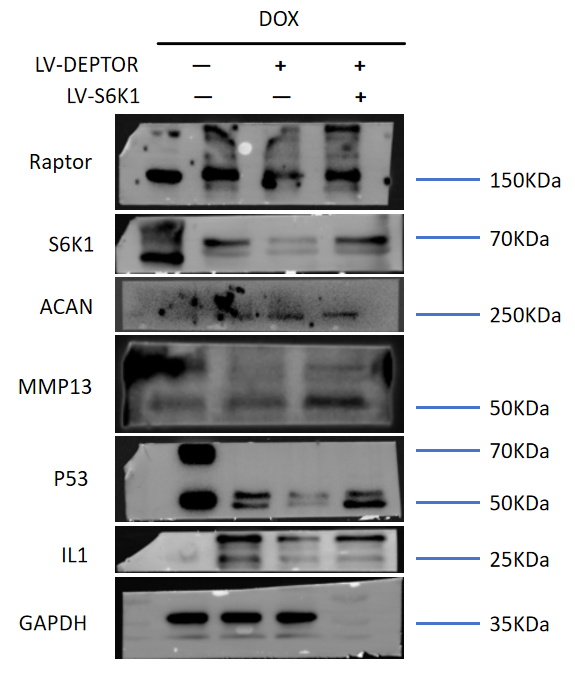
**

**Figure 5C of WB**

**
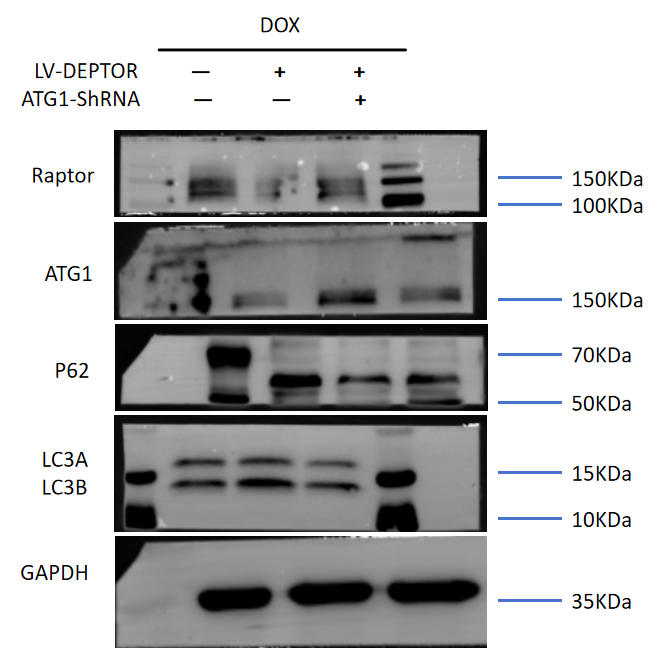
**
